# Supplementary material for: Investigating gene-environment interaction on attention in a double-hit model for Autism Spectrum Disorder
Source: PLoS One. 2024 May 15;19(5):e0299380. doi: 10.1371/journal.pone.0299380 (PMC11095761; doi:10.1371/journal.pone.0299380)
Supplement: S1 File — (DOCX) [file pone.0299380.s001.docx]

| **Task** | **Parameter** | **Delay**  **(p-value)** | **Delay*Genotype**  **(p-value)** | **Delay*Injection**  **(p-value)** | **Delay*Genotype*Injection**  **(p-value)** |
| --- | --- | --- | --- | --- | --- |
| Short-Delay  (Supp. Figure 1) | Accuracy | **0.000** | 0.838 | **0.020** | 0.401 |
|  | Omission | **0.000** | **0.000** | 0.457 | **0.001** |
|  | Perseverative Correct | 0.529 | 0.572 | 0.309 | 0.641 |
|  | Premature Responses | **0.000** | 0.325 | 0.763 | 0.935 |
| Long-Delay  (Supp. Figure 2) | Accuracy | **0.016** | 0.405 | 0.714 | 0.753 |
|  | Omission | **0.000** | 0.418 | 0.516 | 0.902 |
|  | Perseverative Correct | 0.065 | 0.463 | 0.787 | 0.730 |
|  | Premature Responses | **0.000** | 0.244 | 0.188 | 0.158 |
| Distraction  (Supp. Figure 3) | Accuracy | **0.000** | 0.778 | 0.852 | 0.195 |
|  | Omission | **0.000** | 0.090 | 0.410 | 0.877 |
|  | Perseverative Correct | **0.046** | 0.678 | 0.576 | 0.781 |
|  | Premature Responses | **0.000** | 0.115 | **0.002** | 0.537 |

**Supplementary Tables and Figures:**

The statistical values and figures presented below are supplementary findings that more holistically present the involvement of within-subject factors, namely delay and distraction factors during the testing protocol.

**Table 1.** Delay/Distraction main or interaction effects during each respective testing protocol. The p-value was recorded for all testing parameters. N_WT/SALINE_ =10; N_WT/POLY I:C_ =9; N_KO/SALINE_ = 13; N_KO/POLY I:C_ =10.

## Supplementary Figure 1: *Baseline Days to Criterion.* *p < .05; **p < .05; **p < .05. Results are shown as mean ± SEM. N_WT/SALINE_ =10; N_WT/POLY I:C_ =9; N_KO/SALINE_ = 12; N_KO/POLY I:C_ =10.

##

## Supplementary Figure 2: *Short-Delay 5CSRTT Testing.* The parameters A) Omission, B) Perseverative Correct and C) Premature Response are divided across every timepoint of the short-delay paradigm. *p < .05; **p < .05; **p < .05. Results are shown as mean ± SEM. N_WT/SALINE_ =10; N_WT/POLY I:C_ =9; N_KO/SALINE_ = 12; N_KO/POLY I:C_ =10.

## Supplementary Figure 3: *Long-Delay 5CSRTT Testing.* The parameters A) Accuracy, B) Omission and C) Perseverative Correct are divided across every timepoint of the long-delay paradigm. *p < .05; **p < .05; **p < .05. Results are shown as mean ± SEM. N_WT/SALINE_ =10; N_WT/POLY I:C_ =9; N_KO/SALINE_ = 12; N_KO/POLY I:C_ =10.

## Supplementary Figure 4: *Distraction 5CSRTT Testing.* The parameters A) Accuracy, B) Omission and C) Perseverative Correct are divided across every timepoint of the distraction paradigm. *p < .05; **p < .05; **p < .05. Results are shown as mean ± SEM. N_WT/SALINE_ =10; N_WT/POLY I:C_ =9; N_KO/SALINE_ = 12; N_KO/POLY I:C_ =10.
