# Supplementary material for: Investigating gene-environment interaction on attention in a double-hit model for Autism Spectrum Disorder
Source: PLoS One. 2024 May 15;19(5):e0299380. doi: 10.1371/journal.pone.0299380 (PMC11095761; doi:10.1371/journal.pone.0299380)
Supplement: Attachment — Submitted filename: response to reviewers.docx [file pone.0299380.s003.docx]

Reviewer #2: I thank the authors for responding to most of my comments, that manuscript is improved and mush easier to follow. There are some additional results I would still like to see in a supplementary results file, which would not deter from the key findings as you were concerned about in the main manuscript. These include:

1. It would be good to present whether there is a main effect of delay/distraction on any of the factors, just to show how the tests work in general. You have only presented the main effects of genotype and prenatal exposure. When using a repeated measures ANOVA with different within subjects factors of delay or distraction there should be a main effect of these, for example it appears there would be a main effect of increasing delay on premature responses in the long delay test.
For completeness it would be good to list the 2 way interactions as well in supplementary results, i.e. genotype x delay/distraction, poly I:C x delay/distraction and genotype x poly I:C interactions. This could just be in a large table for simplicity.

We have created a table in the supplementary file showing the two- and three-way interactions with the within-subject factors across the three testing paradigms, as requested.

We also want to clarify an error caught in the manuscript. We reran all the analyses when asked to parse the data for the supplementary figures. All data was properly replicated; however, an error was caught in the omission data for the short-delay paradigm. We correctly listed the new values and amended the discussion accordingly to incorporate the significant findings. We apologize for this error and are thankful that it was caught before the paper was finalized.

2. Figures: I would like to see the data split by delay like you have done for Short Delay-Accuracy for all measures in a supplementary file. I agree this would be too much for the main manuscript, however it should be made clear in the figure legends of the main manuscript that the figures include the values for all delays/distractions averaged together, and explain why you have presented it like this (e.g. if there is no significant interaction between delay/distraction and either genotype, poly I:C or both).

We split the data in the supplementary figures for those that were not already parsed in the main manuscript. If they had an interaction effect, we showed that across the different delays/distractions; if there only was a main effect, we collapsed this between-subject factor across the delays/distractions; If there were no significant effects we show a line graph for all four groups to highlight the general trend of each group.

3. The data for number of days to reach criterion which you said you have analysed would be good in a supplementary results file as well.

We added the bar graph that showed the days to criterion for each group. It is appended at the beginning of the supplementary file.
